# How do the year’s seasons and specific weather indices affect physical activity and the patterns of use of public open spaces in the Brazilian context?

**DOI:** 10.1186/s12966-023-01521-2

**Published:** 2023-10-12

**Authors:** Cassiano Ricardo Rech, Carla Elane Silva Godtsfriedt, Gabriel Claudino Budal Arins, Viviane Nogueira de Zorzi, Joris Pazin, Adriano Akira Ferreira Hino, Adalberto Aparecido dos Santos Lopes

**Affiliations:** 1 School of Sports, Federal University of Santa Catarina, R. Eng. Agronômico Andrei Cristian Ferreira, s/n - Trindade, Florianópolis, 88040-900 SC Brazil; 2 State University of Santa Catarina. R. Pascoal Simone, 358 - Coqueiros, Florianópolis, 88080-350 SC Brazil; 3 Medical and Life Science School, Pontifical Catholic University of Parana, Curitiba. R. Imac. Conceição, 1155 - Prado Velho, Curitiba, 80215-901 PR Brazil; 4 Observatory for Urban Health, Federal University of Minas Gerais, Av. Pres. Antônio Carlos, 6627 - Pampulha, Belo Horizonte, 31270-901 MG Brazil

**Keywords:** Natural environment, Parks, Climate, Motor activity

## Abstract

**Background:**

Public open spaces (POS) can offer various resources to promote visitation and engagement in moderate-to-vigorous physical activity (MVPA). However, the influence of seasonal variations and specific meteorological conditions on this relationship remains unclear. Thus, this study aims to investigate the effect of seasonal variations and specific meteorological elements on different days of the week and times of day on POS use and POS-based MVPA in the Brazilian context.

**Methods:**

In 2018, repeated measurements carried out in Southern Brazil used a systematic observation to identify the presence of users in the POS and their engagement in MVPA. The meteorological elements (temperature, thermal sensation, and relative humidity), as well as seasonality (summer, autumn, winter, and spring), were aggregated into the observations.

**Results:**

A total of 19,712 systematic observations were conducted across nine POS. During these observations, a total of 59,354 users were identified. Out of theses, 39,153 (66.0%) were engaged in POS-based MVPA. The presence of users was found to be more frequent during the spring season (38.7%) and on weekends (ranging from 37.6 to 50.1% across seasons). Additionally, user presence was higher in the late afternoon (ranging from 36.4 to 58.2% across seasons) and at higher temperatures with lower relative humidity (p-value < 0.001). Regarding POS-based MVPA, it was more frequent during the winter season (36.4%) and on weekdays (ranging from 73.2 to 79.9% across seasons). Similarly, MVPA was higher in the late afternoon (ranging from 58.3 to 67.5% across seasons) and at lower temperatures and thermal sensations (p-value < 0.005).

**Conclusions:**

Higher presence of users in POS, as well as their visiting, to practice POS-based MVPA, depending on the seasons and specific meteorological elements. By creating infrastructure and conducive conditions, cities can encourage individuals to adopt more active and healthy behaviors. These findings emphasize the importance of designing urban spaces that promote physical activity and contribute to overall well-being.

**Supplementary Information:**

The online version contains supplementary material available at 10.1186/s12966-023-01521-2.

## Background

Green spaces are essential to development of well-planned and sustainable cities by providing suitable areas for physical activity, social interaction, and recreation [1]. The presence of parks, squares, and attractive urban green areas with well-maintained structures encourages their regular use [2]. A diverse urban landscape is closely linked to the promotion of a healthy lifestyle, contributing to an improved quality of life for residents [3, 4]. Moreover, green spaces in urban centers can play a significant role in the conservation of natural areas. They also contribute to enhancing environmental comfort, aiding in urban temperature regulation, while simultaneously reducing energy consumption and pollution rates in cities [5–8].

The utilization of POS is influenced by the conditions of the natural environment [9], including seasonal variations and specific meteorological elements [10]. Extreme temperatures, excessive precipitation leading to rain or snow, strong winds, and low relative humidity are factors that often contribute to reduced utilization of POS and, consequently, less engagement in physical activities in these places [11–13]. It is worth noting that these characteristics may vary and have diverse relationships depending on geographical factors and the time of year.

Regions with harsh winters, such as cities in the United States and Canada, may deter outdoor physical activity [14]. The shorter and colder days lead to a reduction of approximately 10 min in physical activity engagement [12]. Despite the specific climatic conditions in the northern hemisphere these countries offer indoor facilities that enable the relocation of activities during the winter season. However, access to these facilities is often limited to individuals with higher financial resources [15]. During winter, women, those engaging in vigorous-intensity physical activity, and individuals who perceive snow or ice as adverse weather conditions tend to prefer indoor physical activity. Similarly, during summer, older individuals and those who consider rain as an unfavorable weather condition also predominantly opt for indoor physical activity [14].

In general, the utilization of parks is more frequent during the summer season [16], with an increase of approximately 1.5 min per day of physical activity in outdoor spaces for every 5.5-degree Celsius rise in temperature [12]. However, in Sweden, levels of bicycle usage during winter are comparable to those seen in summer, with a high likelihood of commuting to work. This phenomenon can be attributed to cultural factors and the presence of well-developed infrastructure [17]. In Latin America, where tropical and subtropical climates prevail, the effects of seasons and meteorological elements are expected to differ significantly. High temperatures, particularly those exceeding 30 degrees Celsius, tend to limit the use of open public spaces, especially for children and older adults [18].

In recent decades, systematic observation tools have been utilized to assess POS in the context of physical activity [19]. The System for Observing Play and Recreation in Communities (SOPARC) is one such instrument that evaluates user characteristics and activities within POS. It has been widely applied across various regions of the world [20], enabling the collection of climate information through the integration of secondary data sources, including seasonal variations, temperature, thermal sensation, and relative humidity in the assessed areas. However, there is a limited number of studies that have explored seasonality and meteorological elements across different populations while considering contextual factors such as structural characteristics, types of spaces, and commonly performed activities within these places. This scarcity is particularly prominent in Latin American countries. Among 34 studies that utilized SOPARC, only one conducted in North American was addressed the impact of seasonality [21], and when this theme is explored, POS are often not considered as the primary unit of analysis [15]. There is a pressing need for investigations that contextualize and harmonize these factors, facilitating the restructuring of public resources based on prevalent climate conditions in a given region. Such efforts can provide opportunities for physical activity and foster active engagement of the population in utilizing urban spaces.

Thus, this study aims to investigate the effect of seasons variations and specific meteorological elements on a different day of the week and time of day on POS use and.

POS-based MVPA in the Brazilian context.

## Methods

### Study design and location

A repeated systematic observation analysis was conducted, measuring the same locations at four different times of the year. The data were collected during visits to the public open spaces in Florianopolis, Brazil, in 2018. Florianopolis is the capital of the state of Santa Catarina, located in the coastal part of the Southern Region of Brazil. It has a territorial area of 675.409 km^2^ [22] and an estimated population of 492.977 inhabitants; 14.1% aged less than 12 years, 13.6% between 13 and 20 years old, 60.4% between 21 and 59 years old, and 11.5% aged 60 or over [23]. The city has a high Human Development Index - HDI (Florianopolis = 0.847; Brazil = 0.765) [24], and a moderate Social Inequality Index (Gini of 0.5474) [25].

### Public open spaces (POS)

POS are defined as areas within the urban environment that encompass a variety of resources, which are accessible to the community for recreation and fun purposes [26]. To identify all potential POS, we consulted information provided by the Municipal Health Department of the city. A total of 374 sites were identified, geocoded, visited, and evaluated through systematic observation. Among them, 214 POS met the adopted definitions and featured at least one leisure facility for physical activity [2]. From this group, nine POS formed the analytical sample as they fulfilled specific criteria. These criteria included being open to the public, have at least five facilities for physical activity, being available in all regions of the city (north, south, east, continent, and city center), representing different economic strata based on the average per capita income of surrounding residences, and representing areas with higher residential density. Income information (low < R$1886.12 and high > R$1922.67) and residential density [23] were obtained by creating a buffer (circle) with a radius of 500 m from the central point of each POS.

### Identification of the target observation areas

The nine POS selected were visited. The target areas identified as having potential resources for being used for the practice of physical activity were mapped and defined as the unit of analysis of this study. With the purpose of improving the description of the characteristics of the target areas, each one was evaluated through systematic observation by using the Physical Activity Resource Assessment (PARA) [27] in its version adapted for the Brazilian context [28, 29]. PARA is an audit instrument used to identify and evaluate resources available for physical activity, amenities, and incivility items. It utilizes a Likert scale ranging from 0 to 3 points, where: a) ‘0’ represents absence, b) ‘1’ represents presence but with poor quality (not suitable for use), c) ‘2’ represents presence with average quality (usable but requiring improvement), d) ‘3’ represents presence with good quality (in good condition). The instrument calculates the average quality of physical activity structures, the average quality of amenities, and subtracts it from the average quality of incivility [30].

### Observation of the target areas

In order to evaluate the use of the POS, the SOPARC was used. SOPARC was developed and validated to assess the characteristics of park users. In addition, it allows assessing is accessible (e.g. not locked or rented to others), usable (e.g. is not excessively wet or windy), supervised (e.g. by official personnel), organized activities (e.g. team sporting event), and equipped (e.g. removable balls available) in the target area [20].

It is based on momentary time sampling techniques through which systematic and periodic observations of the predetermined areas, named as observation target areas, are carried out [20]. The observation was carried out from left to right by using a code system to measure the presence, gender (male and female), age group (children, adolescents, adults, the elderly), and the physical activity of each individual is coded as sedentary (i.e., lying down, sitting, or standing), walking (moderate), or vigorous in the target area. This process is called scanning. Each scan was performed in a specific way for each group so that it was possible to obtain the levels of physical activity in a specific way for each of these characteristics, thus, with a total of eight scans per target area. In order to facilitate the scanning and recording of information, mechanical counters were used with the data being subsequently transferred to a paper form.

### Training of the observers

For calibration purposes, all nine evaluators who participated in data collection were given 10 h of theoretical training and practical activities in the field by specialists in evaluation through SOPARC [20, 31]. After the training stage, only the observers with adequate reproducibility (ICC > 0.90) were considered able to perform data collection.

### Evaluation periods

All the target areas of the 9 POS were assessed in summer (March), autumn (May), winter (July) and spring (November), 2018. In each season, over the course of a week, each POS was assessed over three days; two days were during the week (Tuesday and Thursday) and one at the weekend (Saturday or Sunday). On each day, the evaluation took place in four moments (07:00 am, 11:00 am, 1:00 pm, and 5:00 pm). Four observations of each target area were carried out in a period of one hour with intervals of approximately 15 min between each observation. There was no data collection in rainy days, given the impact on the reduction of users in the POS.

#### Seasonality and meteorological elements

Seasonality was defined according to the period of the year in which data collection was carried out (summer: from December 21st to March 20th ; autumn: from March 21st to June 20th ; winter: from June 21st to September 20th ; spring: from September 21st to December 20th ). The temperature meteorological elements (in degrees Celsius), thermal sensation (in degrees Celsius) and relative humidity (%) were obtained simultaneously with the systematic observation of each target area at the Information Center for Environmental Resources and Hydrometeorology in Santa Catarina – EPAGRI/CIRAM. The relative humidity is an atmospheric element that refers to the saturation of water present in the air [32]. The thermal sensation is the apparent temperature felt by the exposed skin due to the combination of air temperature and wind speed [32]. All measurements of the atmospheric elements were collected in the initial periods of observation in each target area and were continuously used during the analysis.

All process was approved by the Ethics Committee in Research Involving Human beings of the Federal University of Santa Catarina and adopted the Declaration of Helsinki.

### Statistical analysis

The analysis of the systematic observations on the relative frequency of the users in the POS was applied in order to describe the sample, in addition to assessing the quantitative presence of users in such places. Logistic regression models were created to assess the associations among the seasonality, the presence of the users and their engagement in moderate-to-vigorous physical activity (MVPA). Finally, linear regression was performed to analyze the association among meteorological elements, the presence of users and the practice of MVPA by applying the Durbin-Watson test so as to calculate the autocorrelation in the residuals, as well as the ‘F’ test to identify the model degrees of freedom. The analysis was performed by using SPSS Inc. software (version 25; Chicago, IL) with a significance level of 5%.

## Results

This study assessed nine POS with an average size of 818 m². These POS were situated in an area comprising approximately 2,551.9 residences, with an average per capita income of approximately R$2,004.00. A total of 77 target areas were observed, averaging 8.5 per POS. The availability of resources varied, with exercise stations (100%), such as courts, walking paths, and playgrounds (89.9%), being the most common, followed by lawns (33.3%) and skate tracks/slacklines (11.1%). Most of the target areas were usable (98.3% ), easily accessible (99.8%), and rarely dimly lit (0.3%). The majority of these areas were unoccupied (64.9%) and only a small percentage were supervised (8.9%), organized (0.5%), and equipped (0.3%) at the time of observations.

**Fig. 1:**
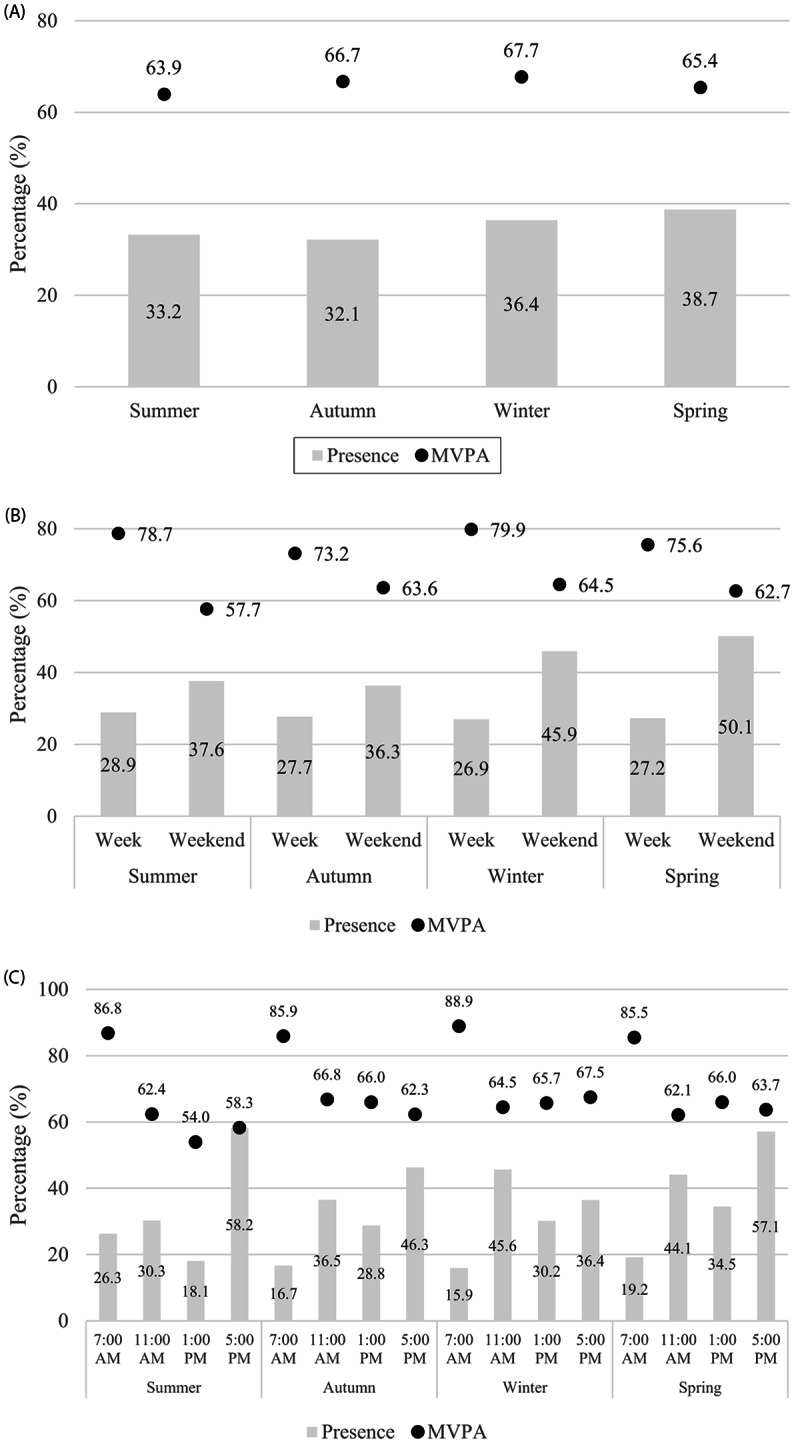
Presence of users in POS and practice of POS-based MVPA according to seasonality in (**A**) seasons of the year, (**B**) weekday and (**C**) time of day. Florianopolis, Brazil, 2018. (n = 9 POS; n = 19,712 observations; n = 59,354 observed users). Note: The analysis of figures ‘**A**’, ‘**B**’ and ‘**C**’ showed a statistically significant association, with a p value of p < 0.001; **A**: association among seasons; **B**: association among the weekdays per season; **C**: association among the periods of the day per season. For more details, see Supplementary Materials 1 and 2; POS: Public open space; MVPA: Moderate-to-vigorous physical activity

A total of 19.712 systematic observations were carried out, and 59.354 users were identified in the POS. A higher frequency of the users was observed in spring (38.1%; OR: 1.29; CI_95%_: 1.19–1.41) and winter (36.4%; OR: 1.14; CI_95%_: 1.05–1.25), when compared to summer. Considering the users engaged in MVPA, it was seen that winter (67.7%; OR: 1.37; CI_95%_: 1.30–1.44), autumn (66.7%; OR: 1.20; CI_95%_: 1.14–1.27) and spring (65.4%; OR: 1.21; CI_95%_: 1.16–1.28) were the most frequent seasons, when compared to summer (Table 1; Fig. 1). Two additional files about Fig. 1 shows this in more detail [see Additional files 1 and 2].

**Table 1:** Association among seasonality, presence and observation of the users who practice POS-based MVPA

Seasonality	Category	Presence^†^ 59,354 (34.0%)		MVPA^‡^ 39,153 (66.0%)	
		%	OR (IC_95%_)	%	OR (IC_95%_)
Season	Summer	33.2	1	63.9	1
	Autumn	32.1	0.94 (0.86 − 1.03)	66.7	1.20 (1.14 − 1.27)*
	Winter	36.4	1.14 (1.05 − 1.25)*	67.7	1.37 (1.30 − 1.44)*
	Spring	38.7	1.29 (1.19 − 1.41)*	65.4	1.21 (1.16 − 1.28)*

**POS**: Public open space; **MVPA**: Moderate-to-vigorous physical activity POS-based; **OR**: Odds ratio; **CI95%**: Confidence interval of 95%; †: model adjusted according to the weekday and period of the day; ‡: model adjusted according to gender, age group, weekday and period of the day*: value of p < 0.001

A higher presence of users was observed on weekends compared to weekdays (p < 0.001) across all seasons. However, engagement in MVPA was more frequent on weekdays (p < 0.001), with a higher frequency of practice in winter (79.9%). The most frequent time slot for user presence across all seasons was the late afternoon, specifically between 5 and 6 p.m. It is worth noting that summer had the highest number of observations (58.2%). Regarding individuals engaged in MVPA, there were more users in the morning, particularly between 7 and 8 a.m., across all seasons, but with a higher frequency in winter (88.9%) - see Fig. 1.

**Fig. 2:**
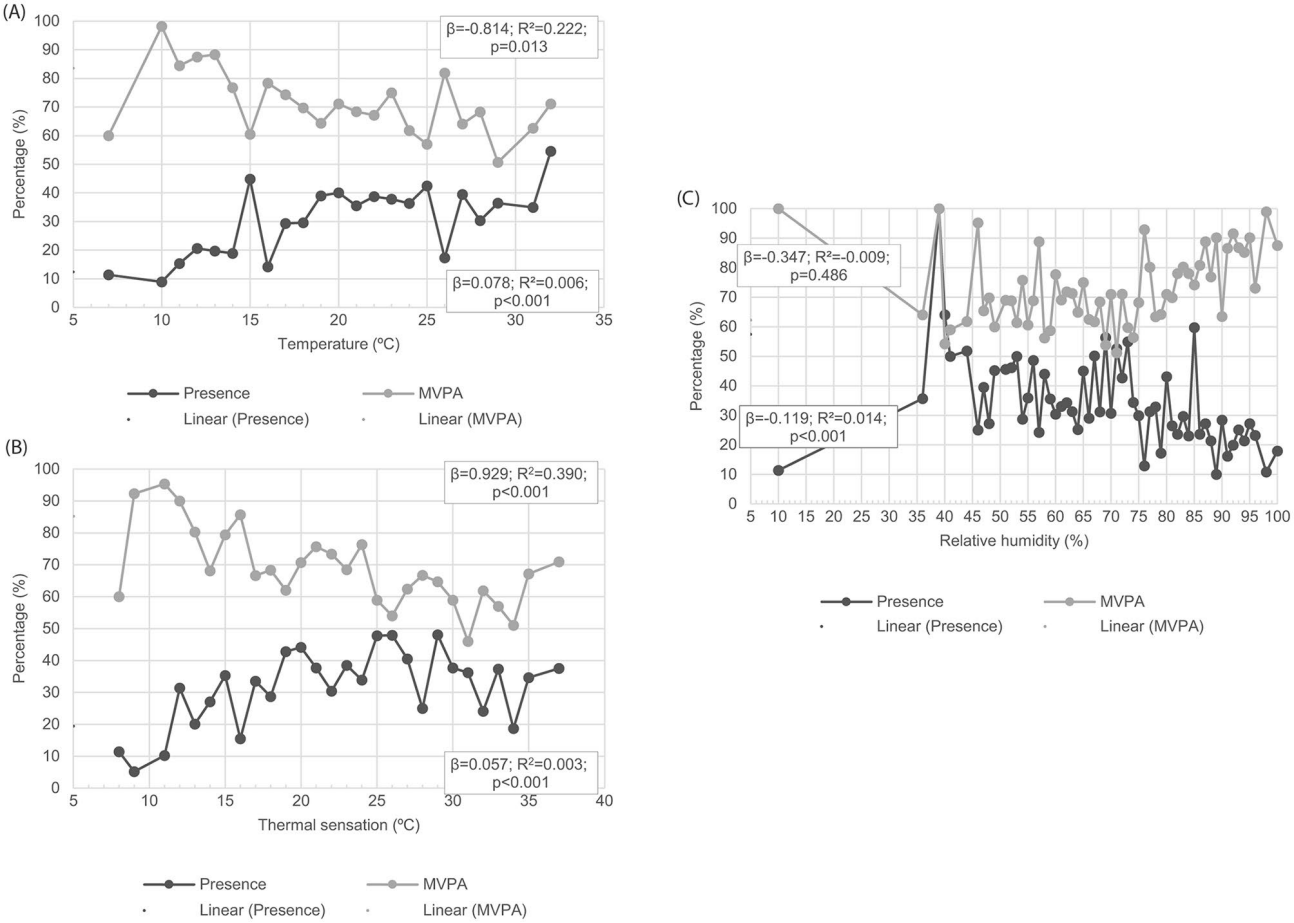
Presence of users in POS and practice of POS-based MVPA according to meteorological elements of (**A**) temperature, (**B**) thermal sensation, and (**C**) relative humidity. Florianopolis, Brazil, 2018. (n = 9 POS; n = 19.712 observations; n = 59.354 observed users). **Note**: The analysis of figures ‘**A**’, ‘**B**’ and ‘**C**’ showed a statistically significant association (p < 0.05), except to relative humidity and MVPA combination; For more details, see Supplementary Material 3; **POS**: Public open space; **MVPA**: Moderate-to-vigorous physical activity

The mean temperature recorded was 20.0 degrees Celsius (SD: 7.0), while the temperature sensation was 22.4 degrees Celsius (SD: 8.4). The relative humidity averaged 68.6% (SD: 18.9). Each degree increase in temperature was associated with presumed increase of 0.07% (p < 0.001) in the number of users in the POS. In contrast, each degree increase in temperature was associated with a decrease of -0.81% in the number of users engaging in MVPA (p = 0.013). In terms of thermal sensation, each degree rises in temperature was associated with a 0.05% increase (p < 0.001) in the number of users in the POS, while each degree increase was associated with a -0.92% decrease (p < 0.001) in the number of users practicing MVPA. Higher relative humidity values were linked to a lower presence of users in the POS (β=-0.119%; p < 0.001), but this was not statistically associated with the number of users engaging in MVPA. Additional information and detailed analysis regarding Fig. 2 can be found in an additional file [see Additional file 3].

## Discussion

The primary objective of this study was to examine the influence of seasons and specific meteorological factors on the utilization of POS and engagement in POS-based MVPA in the Brazilian context. Over the past decade, the prevalence of leisure-time physical activity in Florianopolis, located in southern Brazil, has witnessed an 8% increase [33]. Notably, the presence of high-quality POS throughout the city has contributed significantly to this positive trend [2]. However, there is still substantial room for further improvement, particularly by considering factors that may influence human behavior, such as climate-related features that can impact health, economy, and culture within urban environments [34].

The utilization of a systematic observation method in an environmentally-focused longitudinal study, combined with the monitoring of meteorological elements across the four seasons, represents an innovative approach within the Latin American context. The study’s findings revealed that user presence was more prevalent during spring, on weekends, in the late afternoon, and under higher temperature and thermal sensation conditions, while also being associated with lower relative humidity. Conversely, engagement in MVPA was more frequent during winter, on weekdays, in the late afternoon, and under lower temperature and thermal sensation conditions. These findings highlight the complex interplay between environmental factors and human behavior about physical activity patterns.

A higher number of users in the POS was observed during spring and winter, which aligns with similar findings reported in Canada and the United States [35, 36]. This highlights that seasonal variation has an impact on visitation patterns to outdoor spaces, with temperature being one of the determining factors. The Universal Thermal Climate Index suggests that in subtropical climates characterized by high annual temperature fluctuations and evenly distributed rainfall, thermal comfort in open areas is typically experienced within the range of 15ºC to 24ºC [37]. This finding supports the results of the current study, which identified average temperatures of 17.9 and 22.2 °C during winter and spring, respectively. Given that climate variability has a significant influence on human well-being [38], it is understandable that users prefer seasons with milder temperatures. However, it is crucial to consider the regional temperature variations within each country, particularly in a continental country like Brazil, when devising infrastructure and urban management strategies to mitigate the impact of extreme temperatures on visitation to POS and PA levels due to thermal discomfort [39]. Furthermore, another factor to be considered, especially during periods or in areas with high temperatures, is air pollution, which contributes to lower rates of healthy behaviors in urban environments [40].

The period of visitation to the POS was not found to be related to the different seasons of the year. The majority of users visited the POS on weekends and in the late afternoon, with summer being the season when the highest number of users was observed, particularly between 5 p.m. and 6 p.m. This pattern of visitation is consistent with findings from other contexts, as studies in South Korea and Germany have also identified a high number of users in open spaces during weekends [41, 42]. The preference for weekend visitation can be attributed to people having more leisure time available during this period, as they are less burdened by work tasks. This increased availability allows individuals to engage in activities of their choice and spend time with friends and/or family in open environments, especially in natural surroundings [43]. Additionally, a study conducted in China demonstrated that the time of highest user presence in open spaces is influenced by both seasonal variations and the time of day. In summer, the preferred visitation times were between 8 and 9 a.m. and 6–7 p.m. during which the sunlight intensity is lower, contributing to improved thermal comfort and enjoyment of outdoor activities [44].

During the systematic observations, average temperatures of 20.0ºC, a thermal sensation of 22.4ºC, and a relative humidity of 68.6% were recorded. These values, collected at specific periods, align with the annual average for 2018 in Florianopolis [45]. There is evidence supporting the correlation between these meteorological elements and increased presence of users in open spaces. It was observed that visitation was stimulated when precipitation levels were below 20 mm, average temperatures ranged from 15–16ºC, and humidity was below 60% during spring [1]. Additionally, for trail users, optimal meteorological conditions were found to be maximum daily temperatures between 16 and 22 °C, while temperatures above 31 °C significantly decreased participation in this recreational activities in POS [13]. These findings suggest a linear relationship to some extent between temperature and user engagement. Enhancing the utilization of POS during higher temperatures could be achieved through redesigning the resources within and surrounding the POS, taking into account aspects such as accessibility, connectivity, and the combination of land use to create activity-friendly environments [40, 46].

A significant proportion of users (66%) engaged in MVPA within the POS, highlighting the opportunities that these spaces offer for active lifestyles [47]. Various characteristics, including accessibility, safety for intergenerational coexistence [48], and proximity to open spaces (within 800 m) [47], are likely to encourage users to engage in MVPA. It is noteworthy that weekdays consistently appear more attractive for MVPA, regardless of the season. While literature specifically addressing the relationship between the four seasons and MVPA is limited, similar results have been observed during the European summer [42] and winter [49]. This finding can be partly explained by the combination of proximity between residences and open spaces [42], as well as the presence and quality of green areas and resources that promote MVPA, even considering the demands of a large urban center [49]. Florianopolis, being an island metropolis with 214 POS, 111 of which have a good quality index, featuring comfortable amenities, low incivility, and 53.6% of the equipment for PA in good condition [2], creates favorable conditions for the population living in close proximity to these spaces to engage in MVPA as part of their daily routines. Moreover, the lower proportion of users engaging in MVPA on weekends within the POS may be attributed to the utilization of passive recreation time, contemplation, relaxation, social interactions with friends and family, or engaging in light physical activities such as walking [42, 43].

The findings of this study also revealed that the practice of MVPA in the POS was more common during the early hours of the day and in winter, followed by spring and autumn. This pattern may be attributed to the thermal comfort provided by the specific meteorological conditions associated with each season [14, 50]. A climatological survey conducted in Florianopolis between 1991 and 2020 [45] indicated that the sky was predominantly overcast throughout the year, with the shortest duration of sun exposure occurring during winter (156.7 h), followed by spring (163.9 h), autumn (173.2 h), and summer (185.7 h). Furthermore, the seasons with the fewest rainy days (precipitation > 1 mm) were autumn (average: 18.8 days) and winter (average: 21.2 days), while spring and summer had a higher number of rainy days, averaging around 34 days. These data suggest that meteorological elements and seasonality have an impact on MVPA engagement, particularly during periods of the year characterized by milder temperatures, thermal sensation, and relative humidity. Therefore, it is important to emphasize the consideration of the natural environment [9] in studies exploring human behavior in relation to physical activity [12].

The study findings indicate that an increase in temperature and thermal sensation had a noticeable impact on a small proportion of users engaging in POS-based MVPA. Heat stress has been shown to significantly affect the intensity of physical activity [51, 52]. In regions with a subtropical climate characterized by hot and humid summers, the reduction in basal metabolic rate leads to a decrease in the number of individuals engaging in outdoor activities [51, 53], particularly when the temperature exceeds 33ºC [53]. Conversely, in regions with subarctic climates characterized by harsh winters and short, cold summers [54], as well as in oceanic temperate climates with cool summers and cold winters [55], an increase in temperature may facilitate PA. However, in areas where high temperatures hinder engagement in PA, it is crucial to invest in urban designs adapted to this reality. Measures such as planting trees to provide shade and installing covered drinking fountains and gym equipment can help reduce thermal discomfort, enhance satisfaction, and attract people to engage in PA even during warmer and less humid seasons [51].

We should mention some limitations regarding the interpretation of the results. Firstly, the SOPARC method categorizes PA levels into three broad categories: sedentary behavior, walking for leisure, and MVPA [20]. This categorization does not allow for the assessment of activity duration, making it challenging to conduct a detailed analysis of behavior. In future studies, incorporating objective measures of physical activity could provide a clearer understanding of users’ behavior in the POS [56]. Secondly, it is possible that some individuals entered or left the target area between scans or that their identification was obscured by other users in crowded areas, leading to difficulties in observation during certain periods [20]. Furthermore, we cannot confirm whether POS use is carried out by the same people at different times or are different people. However, it is important to note that the longitudinal design of the study, conducted in a city with a high Human Development Index (HDI) [24] situated in an upper-middle-income country in Latin America [57], provides a comprehensive perspective on the relationships examined.

## Conclusions

Our findings reveal intriguing patterns in the use of POS and engagement in PA in Latin-American context. We discovered that POS visits are more frequent during spring, on weekends, in the late afternoon, and under favorable weather conditions such as higher temperatures and thermal sensations, along with lower humidity. On the other hand, engagement in POS-based MVPA is more common in winter, on weekdays, in the late afternoon, and at lower temperatures and thermal sensations. These insights emphasize the importance of considering the complex interplay between cities, human behavior, and the natural environment when designing sustainable urban spaces. Understanding meteorological elements and seasonality is key to developing effective public policies that enhance POS infrastructure and encourage user presence and active lifestyles.

To create inviting environments for citizens throughout the year, it is essential to prioritize the creation of tree-lined, safe, and comfortable spaces in parks, squares, and urban green areas. These spaces should be equipped with amenities that mitigate extreme weather conditions, ensuring that individuals can engage in PA and enjoy the benefits of a healthy lifestyle on a daily basis. By incorporating these findings into urban planning and design, we can foster active communities, improve public health, and create vibrant and sustainable cities.

### Electronic supplementary material

Below is the link to the electronic supplementary material.

Additional file 1. Association between the seasonal factors and the presence of the users in the POS, stratified by seasons.

Additional file 2. Association between the seasonal factors and the POS-based MVPA stratified according to the seasons.

Additional file 3. Association among meteorological elements with presence of users in POS and the POS-based MVPA.

## Data Availability

The datasets used and/or analyzed during the current study are available from the corresponding author on reasonable request.

## Abbreviations

PARA: Physical Activity Resource Assessment
POS: Public Open Spaces
MVPA: Moderate-to-Vigorous Physical Activity
SOPARC: System for Observing Play and Recreation in Communities
