# Supplementary material for: How do the year’s seasons and specific weather indices affect physical activity and the patterns of use of public open spaces in the Brazilian context?
Source: Int J Behav Nutr Phys Act. 2023 Oct 12;20:124. doi: 10.1186/s12966-023-01521-2 (PMC10571415; doi:10.1186/s12966-023-01521-2)
Supplement: Supplementary file 1 — Additional file 1. Association between the seasonal factors and the presence of the users in the POS, stratified by seasons. [file 12966_2023_1521_MOESM1_ESM.docx]

| **Additional file 1.** Association between the seasonality and the presence of the users in the POS. stratified by seasons. | | | | | | | | | |
| --- | --- | --- | --- | --- | --- | --- | --- | --- | --- |
| **Seasonality** | **Category** | **Summer** | | **Autumn** | | **Winter** | | **Spring** | |
|  |  | % | OR (CI_95%_) | % | OR (CI_95%_) | % | OR (CI_95%_) | % | OR (CI_95%_) |
| Day^†^ | Week | 28.9 | 1 | 27.7 | 1 | 26.9 | 1 | 27.2 | 1 |
|  | Weekend | 37.6 | **1.55 (1.36-1.76)*** | 36.3 | **1.53 (1.35-1.73)*** | 45.9 | **2.52 (2.23-2.86)*** | 50.1 | **2.96 (2.61-3.36)*** |
|  |  |  |  |  |  |  |  |  |  |
| Period^‡^ | 7:00 a.m. | 26.3 | 1 | 16.7 | 1 | 15.9 | 1 | 19.2 | 1 |
|  | 11:00 a.m. | 30.3 | **1.22 (1.02-1.45)*** | 36.5 | **2.88 (2.38-3.48)*** | 45.6 | **4.69 (3.85-5.71)*** | 44.1 | **3.54 (2.94-4.27)*** |
|  | 1:00 a.m. | 18.1 | **0.61 (0.50-0.74)*** | 28.8 | **2.02 (1.66-2.45)*** | 30.2 | **3.14 (2.58-3.83)*** | 34.5 | **2.31 (1.91-2.79)*** |
|  | 5:00 a.m. | 58.2 | **3.96 (3.33-4.70)*** | 46.3 | **4.36 (3.61-5.26)*** | 36.4 | **5.26 (4.33-6.39)*** | 57.1 | **6.21 (5.15-7.50)*** |
|  |  |  |  |  |  |  |  |  |  |
| ***POS****: Public open space;* ***OR****: Odds ratio;* ***CI_95%_:*** *Confidence interval of 95%;* ***†****: model adjusted for the period of the day;* ***‡****: model adjusted for the weekday;* ********: p-value (p<0.001).* | | | | | | | | | |
