# Supplementary material for: How do the year’s seasons and specific weather indices affect physical activity and the patterns of use of public open spaces in the Brazilian context?
Source: Int J Behav Nutr Phys Act. 2023 Oct 12;20:124. doi: 10.1186/s12966-023-01521-2 (PMC10571415; doi:10.1186/s12966-023-01521-2)
Supplement: Supplementary file 2 — Additional file 2. Association between the seasonal factors and the POS-based MVPA stratified according to the seasons. [file 12966_2023_1521_MOESM2_ESM.docx]

| **Additional file 2.** Association between the seasonality and the practice of MVPA in the POS. stratified according to the seasons. | | | | | | | | | |
| --- | --- | --- | --- | --- | --- | --- | --- | --- | --- |
| **Seasonality** | **Category** | **Summer** | | **Autumn** | | **Winter** | | **Spring** | |
|  |  | % | OR (CI_95%_) | % | OR (CI_95%_) | % | OR (CI_95%_) | % | OR (CI_95%_) |
| Day^†^ | Week | 78.7 | 1 | 73.2 | 1 | 79.9 | 1 | 75.6 | 1 |
|  | Weekend | 57.7 | **0.51 (0.46-0.56)*** | 63.6 | **0.78 (0.71-0.85)*** | 64.5 | **0.55 (0.50-0.61)*** | 62.7 | **0.67 (0.61-0.74)*** |
|  |  |  |  |  |  |  |  |  |  |
| Period^‡^ | 7:00 a.m. | 86.8 | 1 | 85.9 | 1 | 88.9 | 1 | 85.5 | 1 |
|  | 11:00 a.m. | 62.4 | **0.36 (0.31-0.42)*** | 66.8 | **0.39 (0.33-0.47)*** | 64.5 | **0.30 (0.25-0.37)*** | 62.1 | **0.35 (0.30-0.42)*** |
|  | 1:00 a.m. | 54.0 | **0.25 (0.21-0.30)*** | 66.0 | **0.39 (0.32-0.47)*** | 65.7 | **0.33 (0.27-0.40)*** | 66.0 | **0.41 (0.35-0.50)*** |
|  | 5:00 a.m. | 58.3 | **0.30 (0.26-0.34)*** | 62.3 | **0.32 (0.27-0.38)*** | 67.5 | **0.34 (0.28-0.42)*** | 63.7 | **0.37 (0.31-0.43)*** |
|  |  |  |  |  |  |  |  |  |  |
| ***POS****: Public open space;* ***MVPA****: Moderate-to-vigorous physical activity;* ***OR:*** *Odds ratio;* ***CI_95%_:*** *Confidence interval of 95%;* ***†****: model adjusted according to gender. age group and period of the day;* ***‡****: model adjusted according to gender. age group and weekday;* ********: p-value (p<0.001).* | | | | | | | | | |
