# Supplementary material for: How do the year’s seasons and specific weather indices affect physical activity and the patterns of use of public open spaces in the Brazilian context?
Source: Int J Behav Nutr Phys Act. 2023 Oct 12;20:124. doi: 10.1186/s12966-023-01521-2 (PMC10571415; doi:10.1186/s12966-023-01521-2)
Supplement: Supplementary file 3 — Additional file 3. Association among meteorological elements with presence of users in POS and the POS-based MVPA. [file 12966_2023_1521_MOESM3_ESM.docx]

| **Additional file 3.** Association among weather indices. the presence of users in POS and the practice of POS-based MVPA. | | | | | | | | | | | | |
| --- | --- | --- | --- | --- | --- | --- | --- | --- | --- | --- | --- | --- |
| **Weather indices** | **Presence**  6.920 (35.1%) | | | | | | **MVPA**  39.153 (66.0%) | | | | | |
|  | β  ^Standardized^ | R^2 Adjusted^ | F | Durbin-Watson | p | CI_95%_ | β | R^2 Adjusted^ | F | Durbin-Watson | p | CI_5%_ |
| Temperature | 0.078 | 0.006 | 119.90 | 1.475 | <0.001 | +0.003 **;** +0.010 | -0.814 | 0.222 | 7.285 | 2.096 | 0.013 | -1.44 **;** -0.18 |
| Thermal sensation | 0.057 | 0.003 | 64.24 | 1.485 | <0.001 | +0.004 **;** +0.006 | -0.929 | 0.390 | 18.241 | 1.428 | <0.001 | -1.37 **;** -0.48 |
| Relative humidity | -0.119 | 0.014 | 280.77 | 1.477 | <0.001 | -0.004 **;** -0.003 | -0.347 | -0.009 | 0.492 | 2.069 | 0.486 | -1.33 **;** +0.64 |
| ***POS****: Public open space;* ***MVPA****: Moderate-to-vigorous physical activity;* ***β****: beta;* ***Durbin-Watson****: residual autocorrelation test;* ***F****: the model degrees of freedom test;* ***p****: significance level;* ***CI_95%_****: confidence interval of 95%.* | | | | | | | | | | | | |
